# Network Models: An Underutilized Tool in Wildlife Epidemiology?

**DOI:** 10.1155/2011/676949

**Published:** 2011-03-10

**Authors:** Meggan E. Craft, Damien Caillaud

**Affiliations:** ^1^Boyd Orr Centre for Population and Ecosystem Heath, College of Medical, Veterinary and Life Sciences, University of Glasgow, Glasgow G12 8QQ, UK; ^2^Section of Integrative Biology, University of Texas, Austin, TX 78712, USA

## Abstract

Although the approach of contact network epidemiology has been increasing in popularity for studying transmission of infectious diseases in human populations, it has generally been an underutilized approach for investigating disease outbreaks in wildlife populations. In this paper we explore the differences between the type of data that can be collected on human and wildlife populations, provide an update on recent advances that have been made in wildlife epidemiology by using a network approach, and discuss why networks might have been underutilized and why networks could and should be used more in the future. We conclude with ideas for future directions and a call for field biologists and network modelers to engage in more cross-disciplinary collaboration.

## 1. Introduction

**Figure 1:**
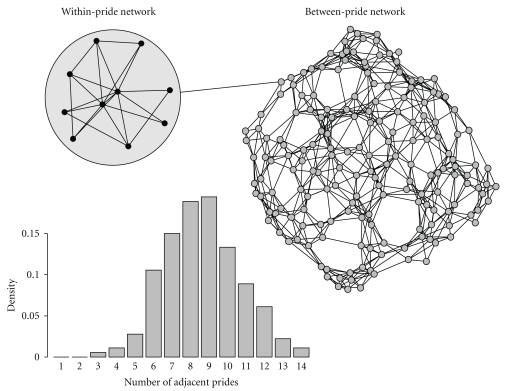
An example of a wildlife network: the Serengeti lion network [4]. In the within-pride network, the nodes (circles) are individuals and edges (lines between circles) are contacts observed on a short time scale (this is a cartoon, not based on data). The between-pride network is derived from behavioral observations of individually known lions as in [4] where nodes represent prides, and edges represent contacts between prides. The histogram represents the degree distribution of the between-pride network.

Conventional methods for studying infectious disease dynamics include a repertoire of modeling techniques: traditional mean field (or Susceptible-Infected-Recovered (SIR) compartmental), metapopulation, lattice-based, reaction-diffusion, and network models [1]. Many of these modeling approaches have been around for decades. The contact network approach, originally developed for applications in the field of statistical physics, has only recently gained in popularity. In network terminology, individuals, or groups of individuals, are defined as *nodes*, connections between those nodes are *edges*, and the number of edges from one node to another is the *degree *(Figure 1). In network epidemiology, diseases spread from node to node following the edges. If the transmission probability along edges is high enough, an epidemic can occur. A very appealing property of networks is their ability to easily depict the complexity of the real world. In particular, the degree distribution captures heterogeneity in transmission among hosts, allowing the disproportionate role of highly connected individuals—superspreaders—to be easily investigated [2, 3]. Networks also often include lists of attributes to nodes or edges that describe between-edge variation in disease transmission or between-host variation in infectiveness or pathogen excretion patterns.

**Figure 2:**
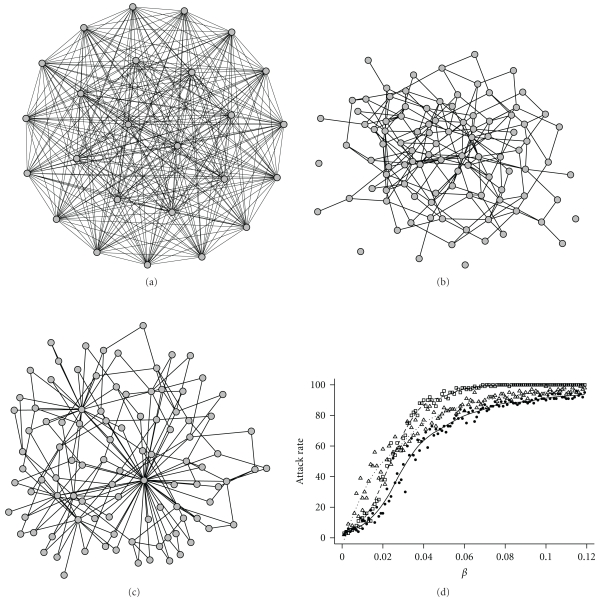
Three examples of contact networks with identical number of nodes (with 100 nodes) and connectedness (where the mean number of effective contacts per node = 4), but different degree distributions. (a) Fully-connected network. Each node has a degree of 99, but a weight of 4/99  =  .0404 is applied to each edge to keep the average connectedness of each node equal to four. Diseases spread through this network in an equivalent way as in a mass action model. For clarity, only 25 nodes out of 100 are represented here. (b) Random network with Poisson degree distribution and mean degree = 4, generated following the Erdos and Renyi model [5]. (c) Scale-free network generated using Barbasi-Albert's preferential attachment algorithm [6], with mean degree = 4 and a power law degree distribution. The network is created by starting with one node and no edges. At each time step, a node is added and connected to two other vertices chosen in proportion to their current degree. This network is characterized by a few highly connected nodes, which may act as superspreaders during epidemics. (d) Stochastic SIR simulations of disease dynamics through the three networks (120 runs per network type). Squares, circles and triangles correspond to networks (a), (b), and (c), respectively. The final epidemic size (attack rate) is represented in relation to the intergroup transmission *β*. The recovery rate is fixed at 0.1. Note that even when the mean connectivity is kept constant, disease impacts vary with network structure.

The network approach is not inherently different from the other modeling tools. It is simply a more general way of representing epidemiological systems. In fact, most alternative models can be considered as particular cases of network model. For example, modeling an epidemic using an SIR compartmental model is equivalent to using a complete network model in which all the nodes are connected to each other (Figure 2(a)). A lattice-based model can also be replaced by a network model in which nodes that are neighbors on the lattice are connected to each other and all nodes have same degree.

Because it offers more flexibility, the network approach can be used to answer new questions and to improve disease control. Since all individuals and all potential transmission paths are represented in the network, it becomes possible to identify individuals or edges that play a key role for disease transmission. Epidemiologists can then propose measures to alter the network in order to prevent or stop disease percolation. For example, vaccinating super-spreading individuals changes the degree distribution, which may be a more efficient way to achieve herd immunity than random vaccination. For these reasons, network techniques have been increasingly used for the study of human diseases [2, 7, 8]. To obtain parameters for these contact network models, populations of humans can rather easily self-report contact data quickly and efficiently through contact tracing or contact diaries [9]; there are also clever ways of using proxies for human disease incidence, such as mining the number of flu-related internet queries [10] or using mobile phone locations as a proxy for human movement or contact-tracing studies [11]. 

Despite the advantages over traditional disease models, networks are still underused for the study of diseases in wild animal populations. In this paper we describe the state of the art for wildlife network modeling, discuss reasons why network models are an underused tool in wildlife epidemiology, and suggest how contact network epidemiology could become more widespread for biologists. For clarity, we restrict our discussion to microparasites with simple life cycles and focus on between-host disease dynamics.

## 2. Current Use of Network Models in Wildlife Epidemiology

This section provides an overview of the current state of wildlife network epidemiology. We first present the main reasons why network models are particularly suited to wildlife epidemiology. We then expose a critical particularity of wildlife epidemiology: the type of data that are collected. Finally, we present recent advances made in the field.

### 2.1. Why Might Network Models Be Preferred to Traditional Modeling Techniques?

Traditional compartmental or metapopulation epidemiological models assume that individuals constituting an epidemiological system can be pooled in a small number of functional groups within which the disease incidence rate is simply proportional to the number of susceptible and infectious individuals. These models are often qualified as “mass-action models”. Within these functional groups, all the individuals are therefore assumed to be epidemiologically identical. The originality of network models resides in their ability to take into account interindividual or intergroup (i.e., internode) variations in epidemiological properties (e.g., degree, infectiveness, recovery rate). With this high resolution, the role played by each individual in the network can be assessed. Since network models capture more heterogeneity among nodes than traditional models, fitted network models can be used to predict the impact of interventions targeting individuals that are critical for disease percolation.

Network models therefore constitute powerful tools to analyze highly heterogeneous epidemiological systems. For example, when the degree distribution is strongly right-skewed, the small number of individuals with the highest degree values tend to be infected very early during the epidemic, and subsequently redistribute the disease to a large number of individuals. These “hub” individuals are then responsible for very high incidence rates at the beginning of the epidemic, which traditional models are unable to predict (see Figure 2). If a mass-action model is used to fit prevalence data collected during this epidemic, the associated goodness-of-fit will be poor, and the estimates of epidemiological parameters will be biased. As explained below, wild animal populations are typical examples of heterogeneous systems and therefore greatly benefit from the network approach.

### 2.2. How Is Wildlife Data Different from Human Data?

 From the epidemiological point of view, wildlife systems differ in four important respects from human systems: (i) the underlying structure of the population, (ii) the tools available to collect data on the network structure, (iii) available epidemiological data, and (iv) potential control options.

First, wild animal populations are often highly structured. Numerous species live in groups, which generally interact nonrandomly. And within a given area, several species susceptible to the same disease can also interact. In such a complex system, the global contact network is modular. It can be decomposed into elements corresponding to the different observation scales: within-group networks (level *n* = 1), between-group networks (*n* = 2), and sometimes higher order level networks like between-species network (*n* = 3). Level-*n* networks (with *n* ≥ 2) can then be considered as metanetworks, that is, networks of networks, with the networks of the level *n*-1 constituting the nodes of the level *n* (Figure 1). Wildlife epidemiologists need to estimate basic structural parameters of their study population in order to know how these different networks are combined together. Basically they need to answer the following questions: do the animals live in groups? If so, what is the group size distribution? How do individuals interact within a group? How do groups interact? Does the disease of interest likely involve several species in the study area? Are there other potentially relevant hierarchical levels, such as subgroups (groups inside groups) or subpopulations (groups of groups)? 

Second, wildlife biologists face multiple challenges when collecting contact data [12–14]. Behavioral observations of animals rarely allow inferring exact, full contact networks, as it is basically impossible to watch all individuals of a population at the same time. The use of indirect measures (through technology) can help this problem, although the number of individuals that can be simultaneously monitored is often limited due to logistical difficulties or the high costs of technology (but see [15] which might have recorded a full network of a study lizard population). More commonly, a representative subset of the study population is generally chosen and then either directly observed using standard behavioral sampling methods or indirectly monitored using biologgers, radio telemetry, mark-recapture, or other methods (for a discussion of methods see Table 1). When choosing a technique to inform a contact model, it is important to take into account whether the species is habituated or not, captive or wild, the local environment of the population (e.g., heavily forested or underwater), the size of the animal, the resolution of the data needed to create a contact network specific to that animal's behavior, the budget, and the sample size needed. It is important not to change the animal's behavior using these techniques, for example through observer presence of timid animals, or heavy tags limiting movement. 

There are a few specific challenges in constructing contact networks from empirical data. (i) Contact networks are normally derived from healthy individuals, and an animal's behavior, and hence the topography of the contact network, might change upon infection. Often it is unknown whether infection would alter the network structure by causing more contacts (e.g., “furious” rabies) or fewer contacts (“dumb” rabies). In this case, a sensitivity analysis could be used to hedge against any changes in contact rates due to infection. (ii) It is quite difficult to define a “contact”; clearly the definition of a contact will depend on the transmission of the pathogen of interest. Is the pathogen sexually transmitted? Aerosol borne? Does it persist in the environment? What is an effective contact? (of course, a contact does not necessarily mean a transmission event.) The best way to get answers to these questions is to do controlled transmission experiments, but this can be ethically challenging, especially for wild animals of conservation concern. (iii) Once a definition of “contact” is created, and a technique chosen to capture these contacts, it is then difficult to measure other types of social interactions for which you are not monitoring. (iv) Despite the recent technological advances allowing the collection of biologically relevant contact data for the majority of a population, how to sample a network is still a problem. For example, technological failures can lead to incomplete networks even if the whole population was successfully tagged, and there are often edge effects with other populations [24]. (v) The type of method used to collect the contact data can influence the properties of the network, hence the infectious period of the disease must be taken into account when choosing a method [27]. Because another behavioral variable is normally being used as a proxy for contact (i.e., proximity data), the raw data collected from these indirect measures does not immediately yield a contact network. But, after adequate processing, it becomes possible to reconstruct contact networks that will not exactly match the actual full network, but will rather have the same statistical, and hence epidemiological, properties. 

Wild animal contact networks also often, if not always, exhibit temporal variation, creating a dynamic network. For example, individuals or groups can migrate to a different area (e.g., reindeer [40], wildebeest [41], birds [42], monarch butterflies [43]), individuals can transfer to a different group (e.g., [44]), and animal societies can fission-fusion (e.g., hyenas [45], chimpanzees [46], bottlenose dolphins [47], elephants [48], lions [49], and guppies [50]). In addition, contact networks can change over long time scales due to demographic processes such as births and deaths. Theoretical studies have shown that the spread of infectious diseases in dynamic networks differs from static networks [51]. Significant changes in contact patterns during the course of an epidemic need to be accounted for, and this data describing contact network dynamics can be obtained using direct observation or technology as listed above and in Table 1.

Third, epidemiological parameters can also be challenging to collect [52]. Incidence can be recorded through passive surveillance operations or direct observation for only the few diseases where wild animals exhibit overt clinical signs (e.g., rabies [53]). However, the majority of wild animals do not show visual signs of disease and most wild animals simply disappear when they die. In the field it is often difficult to detect carcasses, and more worrying, even detect any sort of die-off (e.g., [54]). As another example, out of over 1000 lions suspected to die in a fatal canine distemper virus outbreak in the Serengeti in 1994, only 11 carcasses were recovered from a highly-monitored population [55]. Prevalence data can be collected through active surveillance methods such as serological surveys. Blood can be screened directly for pathogens or indirectly for antibodies to pathogens to provide insight on disease dynamics [56]. Longitudinal surveys are generally the preferred type of serological survey; cross-sectional serological surveys can be misleading because antibodies persist long after the end of the infection [57]. Collecting blood samples is only possible if the study animals can be trapped or darted. It is generally expensive, time-consuming, and potentially risky to the animal. However, in recent years, noninvasive disease screening methods have been developed, such as immunoglobulin dosage in urine and feces (e.g., SIV in chimps [58]) or parasite genotyping in feces (e.g., malaria in great apes [59]). 

In contrast to human diseases, multiple hosts are often involved in wildlife diseases. Human outbreaks often involve animals, but generally only at the very beginning; whereas in wildlife, multiple species are often involved during the entire course of the outbreak. This increases the complexity of building a multihost network, and often it is challenging to have accurate assessments of contact networks from multiple hosts, forcing a fall-back strategy on mean field models [60]. 

Finally, despite constraints to inferring the structure of the contact network and collecting disease data, network models allow us to easily evaluate a wide range of disease control interventions in wildlife populations. In humans, because there are numerous ways to modify human networks, such as school closure, travel warnings, and airport closure in certain cases, public health actions often focus on improving epidemiological surveillance and implementing subsequent vaccination campaigns. In wildlife epidemiology, altering networks is also possible, but in very different ways [61]. For example, oral vaccination baits can occasionally be used with success [62]. Parenteral vaccination can be used for small wild animal populations [63], but is logistically challenging and sometimes considered too invasive. Wildlife contact networks can also be modified by reducing contacts between domestic animals, humans, and wildlife to avoid the spillover to wildlife in the first place—this is a type of quarantine [64–66]. Population density can also be reduced through culling or decreasing birth rates [34, 67]. An important benefit of the network approach is the ability to identify central individuals likely responsible for most transmission events. When those individuals are targeted for intervention purposes, this reduces the number of wildlife needing to be culled or vaccinated, for example.

### 2.3. Recent Progress in Wildlife Network Epidemiology

In the past 5–10 years, wildlife biologists have made solid progress in characterizing contact networks in wildlife populations. Through the use of these contact networks we have been able to address novel questions relating to wildlife and their diseases. For example, superspreading animals have been found in some populations (e.g., deer mice and possums [23, 28]), while they are not obvious in other systems (e.g., Tasmanian devil and African lion populations [4, 24]). The Tasmanian devil network was found to be one giant component, meaning that the whole endangered population is threatened by a novel infectious cancer [24]. Well-connected individuals were more likely to be infected in some wildlife populations (brushtail possums, sleepy lizards, skinks, and bumble-bees [15, 29, 32, 39]) but not in others (meerkats [16]). In a study of possums, density was found to be uncorrelated with contact rates [25]. In contrast, abundance thresholds above which disease can spread (percolation thresholds) have been identified in gerbils with plague [68] and in multihost plague systems of mice and prairie dogs [69]. With networks, researchers were able to distinguish spatial patterns of disease spillover from epidemic waves [17]. Temporal changes in contact patterns were also identified as critical for the spread of respiratory diseases in wild chimpanzees [70]. Issues of different spatial scales have been tackled with networks, specifically the relative importance of local versus long range transmission events in driving disease spread [68]. Finally, multilevel network models have been developed and successfully applied to tuberculosis transmission between badgers groups and cows [26]. For a more extensive list of insights we refer to Table 1 of a recent review [71].

## 3. Despite These Advances, Why Are Network Models Underused in Wildlife Epidemiology?

The network approach might need a public relations campaign in the literature. Networks have been used in other fields like statistical physics for decades, yet have only in the past 10 years really taken off with the human epidemiology literature, and are now at the cutting edge of wildlife epidemiology. Contact networks models are likely *not well-known* in the wildlife community. The number of studies using this approach is still relatively limited, but of those studies combining networks and wildlife, they often get published in high profile journals—potentially indicating that wildlife network epidemiology is still in its infancy. 

Second, the network literature, especially in the physics literature, is quite hard to grasp and at first sight, may seem *complicated* for the field biologist. The analytical treatment of network epidemiological models is however only slightly more difficult than solving systems of differential equations of mass action models. A few articles are notable for presenting the mathematics of network models in an accessible way for biologists [72–74]. However, finding analytical solutions is not always necessary. Agent-based stochastic simulations can be good alternatives and are relatively easy to implement. They are particularly interesting to model complex systems that have a lot of parameters and hence cannot be described by closed form equations. Unfortunately there is limited network software with easy-to-use graphical user interfaces implementing these methods, and often people program their own network simulation models (Table 2). Coding using basic programming languages (e.g., R, Python or NetLogo) is often a disparate skill set from a field biologist who successfully collects contact and epidemiological data on wild populations.

Third, networks are indeed *data intensive*, and wildlife systems are unfortunately often data limited. Individual-level data can be expensive and time-consuming to collect [71]. For example, in constructing a contact network for a population of Serengeti lions, only 36 pride-to-pride contacts were observed per 1294 hours of daylight observation over a 3 year time period [4]. In addition, wild animals cannot be continuously observed, and dealing with gaps and missing data is often challenging (see Section 4).

Finally, contact networks are inferred from contact data collected for a specific species, for a specific ecosystem, and for a specific period of time. Therefore it can be *difficult to generalize *epidemiological results obtained with a network model to other circumstances (e.g., [4]).

## 4. What Can Be Done to Dispel Doubts about Networks?

Network approaches need to become better *known* and more accessible. Wildlife epidemiologists should be encouraged to promote their network approach at meetings and in journals that have not normally embraced a network approach. More training sessions such as SISMID (University of Washington) or INSNA's workshops at Sunbelt would be useful. A formal comparison between network and mean-field models would also help spread the word. Currently, there are few papers comparing the performance of mean-field and network models. Although scientists might have tried multiple modeling approaches during the course of the study, normally only one approach is published. Mass action models normally work “well enough,” but we are unaware of any formal quantitative comparison of the pros and cons of using mean-field versus network models for a range of empirical and theoretical systems. 

It is likely that network models are only going to get more *complex*: they will include more parameters and variables. “Complexity” is an intrinsic, objective property of a model. It is not necessarily synonymous with “complicated”—a subjective judgment of the difficulty of the modeling task. For example, it is important to note that stochastic agent-based modeling handles very complex models but is generally not complicated. In the last few years, biologists have increasingly used these types of models to investigate respective effects of different variables on biological phenomenon. Several software and user-friendly computer languages are particularly suited to develop network epidemiological agent-based models (Table 2). It has even become possible to fit such models to field data. The recent developments in approximate Bayesian computation (ABC), a set of methods initially developed by population geneticists, greatly facilitate agent-based model fitting [84–86]. We would like to attract the attention of epidemiologist to these methods, which we believe will be used extensively in the future. 

Technology is helping to bridge the gap between *data-intensive* network models, and the challenges inherent in collecting contact data. There has been a burst of new technology such as satellite GPS radio telemetry, proximity data loggers, camera and video traps, tracking, proximity data radio collars, powder marking, PIT tags, and antennae, and capture-mark-recapture (Table 1). These methods almost always collect data at discrete time intervals. Ignoring the gaps inherent to these datasets can lead to biased estimates of contact network or epidemiological parameters. For example, if an animal is observed susceptible at time *t* and reobserved infected 10 days later, should we assume that it became infected on the first day, on the last day, or maybe after five days? The answer is that none of these assumptions is necessary. At least three statistical methods can be used to deal with this uncertainty. First, survival analyses, that were initially developed to estimate survival rates using date-of-last-observation data can also be applied, for example, to estimate rates of seroconversion. One simply has to assume that seroconversion is equivalent to death [87]. Second, multistate capture-mark-recapture (CMR) models have proven very useful to estimate animal migration rates, survival rates, and rates of change of individual state (for example, states S, I or R). Although user-friendly software exists to fit CMR models (Table 2), these models could be more broadly used in wildlife epidemiology, both to estimate network parameters and epidemiological parameters. Third, agent-based models, coupled with ABC fitting procedure, can easily circumvent the problem of missing data [84–86]. Our purpose here is to attract the attention of epidemiologists to these methods rather than to describe them in detail, so we encourage interested readers to consult the references cited.

An exciting and useful push for future directions would be to develop theoretical advances for network models that allow us to develop “universal principles”. As stated above, current network models are generally inferred from contact data. But one generally does not know what rules govern the establishment of contact patterns. Understanding these rules, in particular how ecological variables such as food resource distribution, distribution of conspecifics, and climate influence contact patterns, would allow identifying universal principles governing networks' structure. It would then become possible to extrapolate these mechanistic models to other populations, areas, or time periods.

Network epidemiological modeling is by essence interdisciplinary. This is even more pertinent to wildlife network epidemiology, because new fields such as behavioral ecology, capture-mark-recapture, and advanced statistics are combined. Collaborative work is an efficient way to do network modeling. Field biologists know their system, and know how to collect data, while theoreticians can work on the hardcore modeling aspects. We would like to promote better collaboration between modelers and field biologists. Modelers may need to be seen as more approachable by field biologists. Importantly, we believe collaborations should take place at all stages of epidemiological studies, from the design of the data collection protocol to the end of the modeling stage.

## 5. Conclusion

Network models have promising applications in the field of wildlife epidemiology. Although using this approach requires some substantial training, the learning curve is not as steep as it seems, and several software and interpreted computer languages have been developed that will make this step easier. In any case, we strongly believe that the benefits far outweigh these costs. The number of applications of network models in wildlife epidemiology is already broad, and will keep increasing. New application domains beg to be explored. For example, network models are well-suited to combine network and genetic data, potentially for viral diseases such as feline immunodeficiency virus and simian immunodeficiency virus. Contact network epidemiology using directed networks (where there is stronger transmission in one direction) has been applied to animals using the same resting spots for indirectly transmitted pathogens [15, 29], and could be expanded to fresh water organisms because river networks are easy to map, have a good spatial component, and pathogens might travel downstream.

We feel that developing collaboration between field biologists and network modelers will be a key factor bringing advances to wildlife epidemiology. We need to become more multidisciplinary and cross disciplinary [14, 88].

## Figures and Tables

**Table 1:** Direct and indirect techiques that could be used to collect contact network data on wildlife populations and selected examples using these techniques.

**Technique**	**Useful for which type of species?**	**Comments**	**Selected references**
*Direct*			

Behavioral observations of known individuals	Diurnal habituated animals that can be easily observed (not cryptic species)	Potentially a “gold standard” for contact networks (multiple types of social interactions can be recorded); labor intensive	[16–19]

Viewpoint scanning	Visible animals active during the day; open habitat (not cryptic species)	Allows between-species observations at replicable sites; labor intensive yet incomplete observations	[20]

*Indirect*			

Biologging	Easily captured and handled individuals	Population needs to be saturated with detectors; excellent resolution of proximity data although proximity does not mean contact; continuous time record; cannot distinguish between types of close contacts (e.g., fighting versus mating)	[21]
Biologging: animal-borne acoustic proximity receiver	Marine mammals	Need to handle animal to retrieve device; good between-animal resolution	[22]
Biologging: PIT (Passive Integrated Transponder) tags	Useful for small mammals	Good data on duration of presence/absence of marked individuals at specific places (e.g., supplemented foraging sites) equipped with PIT loggers; approximation of contacts	[23]
Biologging: proximity data loggers/collars	Medium to large animals	measure frequency and duration of contact; complete temporal data; need to recover loggers	[24–26]

Capture-mark-recapture	Easily captured and handled individuals	A contact is defined as occupying same area during same period of time; good for capturing movement/dispersal data, not good at capturing within-group contacts	[27–31]

Direct manipulation	Captive populations of common animals	Great for repeatable experiments on experimentally infected individuals to measure transmission, but does this reflect contact patterns in wild?	[32, 33]

GPS recorders	Easily captured and handled medium to large individuals	Need recorders on all individuals in select area; if recorders are synced well, excellect contact data for the time the GPS takes point (with spotty coverage in between). Maybe local avoidance happens but would be undetected?	[15]

Powder marking	Easily trapped and handled individuals	Gives good contact data if contacts involve direct phyical contact; can only monitor a few indivuals at a time due to contstraints on the number of powder colors	[23]

Radio telemetry	Handled individuals, not good for very small individuals	Contact defined as occupying same area during same period of time. Good indicator of (i) scale of interaction but gives coarse resolution of a “contact”, (ii) mixing between groups of animals, but not within groups and (iii) den-sharing contacts. Presence of fieldworkers may alter behavior.	[27, 34–36]

Trapping and bait marking	Easily trapped and handled individuals who use latrines to mark territories	Good data on home range overlap and intergroup movement rates	[37]

Video tracking from animal's perspective	Animal must be able to be caught and wear something like a video backpack	Great contact data from individual perspective	[38]

Video trapping from fixed perspective (automated)	Social insects that can be individually tagged and the group monitored	Great resolution of contact data; software records duration and frequency of contacts	[39]

**Table 2:** Selected list of free software packages that can be used in wildlife network epidemiology.

Category name	Type	Programming skills required?	Comments	Ref./Download location
*Capture-mark-recapture*				

M-Surge	Application	No	Windows platform	[75]/http://www.cefe.cnrs.fr/BIOM/logiciels.htm
MARK	Application	No	Windows platform	[76]/http://warnercnr.colostate.edu/~gwhite/mark/mark.htm

*Network simulation/analysis*				

Pajek	Application	No	Analysis of large networks	[77]/http://vlado.fmf.uni-lj.si/pub/networks/pajek/
igraph	R Package	Basic	Used for all network visualizations in this article	[78]/http://www.r-project.org/
sna	R package	Basic	Other related R packages available on http://statnet.org/	[79]/http://www.r-project.org/
network	R Package	Basic	Other related R packages available on http://statnet.org/	[80]/http://www.r-project.org/
ClustRNet	Python package	Basic	Simulates network with variable clustering degree	[81]/http://sbansal.com/ClustRNet/
NetLogo	Interpreted language	Basic	Cross-platform	[82]/http://ccl.northwestern.edu/netlogo/

*Epidemiological modelling*				

EpiFire (GUI)	Application	No	Cross-platform	unpublished/http://sourceforge.net/projects/epifire/
NetLogo	Interpreted language	Basic	Cross-platform	[82]/http://ccl.northwestern.edu/netlogo/
R	Interpreted language	Basic	Cross-platform	[83]/http://www.r-project.org/
Python	Interpreted language	Basic	Cross-platform	http://www.python.org/
EpiFire (API)	C++ library	Yes (C/C++)	Includes network simulation capabilities	unpublished/http://sourceforge.net/projects/epifire/

## References

[1] Keeling MJ, Rohani P (2008). *Modeling Infectious Diseases in Humans and Animals*.

[2] Meyers LA, Pourbohloul B, Newman MEJ, Skowronski DM, Brunham RC (2005). Network theory and SARS: predicting outbreak diversity. *Journal of Theoretical Biology*.

[3] Lloyd-Smith JO, Schreiber SJ, Kopp PE, Getz WM (2005). Superspreading and the effect of individual variation on disease emergence. *Nature*.

[4] Craft ME, Volz E, Packer C, Meyers LA Disease transmission in territorial populations: the small-world network of Serengeti lions.

[5] Erdos P, Renyi A (1959). On random graphs. *Publicationes Mathematicae*.

[6] Barabási AL, Albert R (1999). Emergence of scaling in random networks. *Science*.

[7] Pourbohloul B, Meyers LA, Skowronski DM, Krajden M, Patrick DM, Brunham RC (2005). Modeling control strategies of respiratory pathogens. *Emerging Infectious Diseases*.

[8] Bansal S, Pourbohloul B, Meyers LA (2006). A comparative analysis of influenza vaccination programs. *PLoS Medicine*.

[9] Keeling MJ, Eames KTD (2005). Networks and epidemic models. *Journal of the Royal Society Interface*.

[10] Ginsberg J, Mohebbi MH, Patel RS, Brammer L, Smolinski MS, Brilliant L (2009). Detecting influenza epidemics using search engine query data. *Nature*.

[11] King DA, Peckham C, Waage JK, Brownlie J, Woolhouse MEJ (2006). Infectious diseases: preparing for the future. *Science*.

[12] Wey T, Blumstein DT, Shen W, Jordán F (2008). Social network analysis of animal behaviour: a promising tool for the study of sociality. *Animal Behaviour*.

[13] Krause J, Croft DP, James R (2007). Social network theory in the behavioural sciences: potential applications. *Behavioral Ecology and Sociobiology*.

[14] McCallum H (2009). Six degrees of *Apodemus* separation. *Journal of Animal Ecology*.

[15] Leu ST, Kappeler PM, Bull CM (2010). Refuge sharing network predicts ectoparasite load in a lizard. *Behavioral Ecology and Sociobiology*.

[16] Drewe JA (2010). Who infects whom? Social networks and tuberculosis transmission in wild meerkats. *Proceedings of the Royal Society B*.

[17] Craft ME, Volz E, Packer C, Meyers LA (2009). Distinguishing epidemic waves from disease spillover in a wildlife population. *Proceedings of the Royal Society B*.

[18] Altmann J (1974). Observational study of behavior: sampling methods. *Behaviour*.

[19] de Menezes MA, Baird RW, Lusseau D, Guimarães P, Guimarães PR, dos Reis SF (2007). Vulnerability of a killer whale social network to disease outbreaks. *Physical Review E*.

[20] Richomme C, Gauthier D, Fromont E (2006). Contact rates and exposure to inter-species disease transmission in mountain ungulates. *Epidemiology and Infection*.

[21] Prange S, Jordan T, Hunter C, Gehrt SD (2006). New radiocollars for the detection of proximity among individuals. *Wildlife Society Bulletin*.

[22] Guttridge TL, Gruber SH, Krause J, Sims DW (2010). Novel acoustic technology for studying free-ranging shark social behaviour by recording individuals’ interactions. *PLoS ONE*.

[23] Clay CA, Lehmer EM, Previtali A, St. Jeor S, Dearing MD (2009). Contact heterogeneity in deer mice: implications for Sin Nombre virus transmission. *Proceedings of the Royal Society B*.

[24] Hamede RK, Bashford J, McCallum H, Jones M (2009). Contact networks in a wild Tasmanian devil (*Sarcophilus harrisii*) population: using social network analysis to reveal seasonal variability in social behaviour and its implications for transmission of devil facial tumour disease. *Ecology Letters*.

[25] Ji W, White PCL, Clout MN (2005). Contact rates between possums revealed by proximity data loggers. *Journal of Applied Ecology*.

[26] Böhm M, Hutchings MR, White PCL (2009). Contact networks in a wildlife-livestock host community: identifying high-risk individuals in the transmission of bovine TB among badgers and cattle. *PLoS ONE*.

[27] Perkins SE, Cagnacci F, Stradiotto A, Arnoldi D, Hudson PJ (2009). Comparison of social networks derived from ecological data: implications for inferring infectious disease dynamics. *Journal of Animal Ecology*.

[28] Porphyre T, Stevenson M, Jackson R, McKenzie J (2008). Influence of contact heterogeneity on TB reproduction ratio R in a free-living brushtail possum Trichosurus vulpecula population. *Veterinary Research*.

[29] Godfrey SS, Bull CM, James R, Murray K (2009). Network structure and parasite transmission in a group living lizard, the gidgee skink, Egernia stokesii. *Behavioral Ecology and Sociobiology*.

[30] Lebreton JD, Burnham KP, Clobert J, Anderson DR (1992). Modeling survival and testing biological hypotheses using marked animals: a unified approach with case studies. *Ecological Monographs*.

[31] Pollock KH, Nichols JD, Brownie C, Hines JE (1990). Statistical inference for capture-recapture experiments. *Wildlife Monographs*.

[32] Corner LAL, Pfeiffer DU, Morris RS (2003). Social-network analysis of Mycobacterium bovis transmission among captive brushtail possums (Trichosurus vulpecula). *Preventive Veterinary Medicine*.

[33] Naug D (2008). Structure of the social network and its influence on transmission dynamics in a honeybee colony. *Behavioral Ecology and Sociobiology*.

[34] Ramsey D, Spencer N, Caley P (2002). The effects of reducing population density on contact rates between brushtail possums: implications for transmission of bovine tuberculosis. *Journal of Applied Ecology*.

[35] Gompper ME, Wright AN (2005). Altered prevalence of raccoon roundworm (Baylisascaris procyonis) owing to manipulated contact rates of hosts. *Journal of Zoology*.

[36] Cross PC, Lloyd-Smith JO, Bowers JA, Hay CT, Hofmeyr M, Getz WM (2004). Integrating association data and disease dynamics in a social ungulate: bovine tuberculosis in African buffalo in the Kruger National Park. *Annales Zoologici Fennici*.

[37] Vicente J, Delahay RJ, Walker NJ, Cheeseman CL (2007). Social organization and movement influence the incidence of bovine tuberculosis in an undisturbed high-density badger Meles meles population. *Journal of Animal Ecology*.

[38] Bluff LA, Rutz C (2008). A quick guide to video-tracking birds. *Biology Letters*.

[39] Otterstatter MC, Thomson JD (2007). Contact networks and transmission of an intestinal pathogen in bumble bee (*Bombus impatiens*) colonies. *Oecologia*.

[40] Sharma S, Couturier S, Côté SD (2009). Impacts of climate change on the seasonal distribution of migratory caribou. *Global Change Biology*.

[41] Holdo RM, Holt RD, Fryxell JM (2009). Opposing rainfall and plant nutritional gradients best explain the wildebeest migration in the serengeti. *The American Naturalist*.

[42] Rappole JH, Derrickson SR, Hubálek Z (2000). Migratory birds and spread of West Nile virus in the Western Hemisphere. *Emerging Infectious Diseases*.

[43] Brower LP (1995). Understanding and misunderstanding the migration of the monarch butterfly (Nymphalidae) in North America: 1857–1995. *Journal - Lepidopterists’ Society*.

[44] Sterck EHM, Watts DP, van Schaik CP (1997). The evolution of female social relationships in nonhuman primates. *Behavioral Ecology and Sociobiology*.

[45] Smith JE, Kolowski JM, Graham KE, Dawes SE, Holekamp KE (2008). Social and ecological determinants of fission-fusion dynamics in the spotted hyaena. *Animal Behaviour*.

[46] Lehmann J, Boesch C (2004). To fission or to fusion: effect of community size on wild chimpanzee (Pan troglodytes versus) social organisation. *Behavioral Ecology and Sociobiology*.

[47] Connor RC, Wells R, Mann J, Read A, Mann J, Connor R, Tyack P, Whitehead H (2000). The bottlenose dolphin: social relationships in a fission-fusion society. *Cetacean Societies: Field Studies of Whales and Dolphins*.

[48] Wittemyer G, Douglas-Hamilton I, Getz WM (2005). The socioecology of elephants: analysis of the processes creating multitiered social structures. *Animal Behaviour*.

[49] Schaller GB (1972). *The Serengeti Lion; A Study of Predator-Prey Relations*.

[50] Croft DP, Krause J, James R (2004). Social networks in the guppy (Poecilia reticulata). *Proceedings of the Royal Society B*.

[51] Volz E, Meyers LA (2009). Epidemic thresholds in dynamic contact networks. *Journal of the Royal Society Interface*.

[52] Cleaveland S, Packer C, Hampson K, Sinclair ARE, Mduma S, Fryxell J (2008). The multiple roles of infectious diseases in the Serengeti ecosystem. *Serengeti III: Human Impacts on Ecosystem Dynamics*.

[53] Lembo T, Hampson K, Haydon DT (2008). Exploring reservoir dynamics: a case study of rabies in the Serengeti ecosystem. *Journal of Applied Ecology*.

[54] Caillaud D, Levréro F, Cristescu R (2006). Gorilla susceptibility to Ebola virus: the cost of sociality. *Current Biology*.

[55] Roelke-Parker ME, Munson L, Packer C (1996). A canine distemper virus epidemic in Serengeti lions (Panthera leo). *Nature*.

[56] Packer C, Altizer S, Appel M (1999). Viruses of the Serengeti: patterns of infection and mortality in African lions. *Journal of Animal Ecology*.

[57] Cleaveland S, Mlengeya T, Kaare M (2007). The conservation relevance of epidemiological research into carnivore viral diseases in the Serengeti. *Conservation Biology*.

[58] Santiago ML, Lukasik M, Kamenya S (2003). Foci of endemic simian immunodeficiency virus infection in wild-living eastern chimpanzees (Pan troglodytes schweinfurthii). *Journal of Virology*.

[59] Prugnolle F, Durand P, Neel C (2010). African great apes are natural hosts of multiple related malaria species, including Plasmodium falciparum. *Proceedings of the National Academy of Sciences of the United States of America*.

[60] Craft ME, Hawthorne PL, Packer C, Dobson AP (2008). Dynamics of a multihost pathogen in a carnivore community. *Journal of Animal Ecology*.

[61] Delahay RJ, Smith GC, Hutchings MR (2009). *Management of Disease in Wild Mammals*.

[62] Cross ML, Buddle BM, Aldwell FE (2007). The potential of oral vaccines for disease control in wildlife species. *The Veterinary Journal*.

[63] Haydon DT, Randall DA, Matthews L (2006). Low-coverage vaccination strategies for the conservation of endangered species. *Nature*.

[64] Rwego IB, Isabirye-Basuta G, Gillespie TR, Goldberg TL (2008). Gastrointestinal bacterial transmission among humans, mountain gorillas, and livestock in Bwindi Impenetrable National Park, Uganda. *Conservation Biology*.

[65] Köndgen S, Kühl H, N’Goran PK (2008). Pandemic human viruses cause decline of endangered great apes. *Current Biology*.

[66] Osofsky SA, Cleaveland S, Karesh WB (2005). *Conservation and Development Interventions at the Wildlife/Livestock Interface: Implications for Wildlife, Livestock, and Human Health*.

[67] Caughley G, Pech R, Grice D (1992). Effect of fertility control on a population’s productivity. *Wildlife Research*.

[68] Davis S, Trapman P, Leirs H, Begon M, Heesterbeek JAP (2008). The abundance threshold for plague as a critical percolation phenomenon. *Nature*.

[69] Salkeld DJ, Salathé M, Stapp P, Jones JH (2010). Plague outbreaks in prairie dog populations explained by percolation thresholds of alternate host abundance. *Proceedings of the National Academy of Sciences of the United States of America*.

[70] Kuehl HS, Elzner C, Moebius Y, Boesch C, Walsh PD (2008). The price of play: self-organized infant mortality cycles in chimpanzees. *PLoS ONE*.

[71] Tompkins DM, Dunn AM, Smith MJ, Telfer S (2011). Wildlife diseases: from individuals to ecosystems. *Journal of Animal Ecology*.

[72] Newman MEJ (2002). Spread of epidemic disease on networks. *Physical Review E*.

[73] Newman MEJ, Strogatz SH, Watts DJ (2001). Random graphs with arbitrary degree distributions and their applications. *Physical Review E*.

[74] Meyers LA, Newman MEJ, Pourbohloul B (2006). Predicting epidemics on directed contact networks. *Journal of Theoretical Biology*.

[75] Choquet R, Reboulet AM, Pradel R, Gimenez O, Lebreton JD (2004). M-SURGE: new software specifically designed for multistate capture-recapture models. *Animal Biodiversity and Conservation*.

[76] White GC, Burnham KP (1999). Program MARK: survival estimation from populations of marked animals. *Bird Study*.

[77] Batagelj V, Mrvar AP, Jünger M, Mutzel P (2003). Analysis and visualization of large networks. *Graph Drawing Software*.

[78] Csardi G, Nepusz T (2006). The igraph software package for complex network research. *InterJournal Complex Systems*.

[79] Handcock MS, Hunter DR, Butts CT, Goodreau SM, Morris M (2008). statnet: software tools for the representation, visualization, analysis and simulation of network data. *Journal of Statistical Software*.

[80] Butts CT (2008). network: a package for managing relational data in R. *Journal of Statistical Software*.

[81] Bansal S, Khandelwal S, Meyers LA (2009). Exploring biological network structure with clustered random networks. *BMC Bioinformatics*.

[82] Wilensky U (1999). *NetLogo: Center for Connected Learning and Computer-Based Modeling*.

[83] R Development Core Team (2010). *R: A Language and Environment for Statistical Computing*.

[84] Toni T, Welch D, Strelkowa N, Ipsen A, Stumpf MPH (2009). Approximate Bayesian computation scheme for parameter inference and model selection in dynamical systems. *Journal of the Royal Society Interface*.

[85] Wegmann D, Leuenberger C, Excoffier L (2009). Efficient approximate Bayesian computation coupled with Markov chain Monte Carlo without likelihood. *Genetics*.

[86] Leuenberger C, Wegmann D (2010). Bayesian computation and model selection without likelihoods. *Genetics*.

[87] Hagan H, Thiede H, Des Jarlais DC (2004). Hepatitis C virus infection among injection drug users: survival analysis of time to seroconversion. *Epidemiology*.

[88] Haydon DT (2008). Cross-disciplinary demands of multihost pathogens. *Journal of Animal Ecology*.

